# DNA methylation signatures in cord blood associated with birthweight are enriched for dmCpGs previously associated with maternal hypertension or pre-eclampsia, smoking and folic acid intake

**DOI:** 10.1080/15592294.2021.1908706

**Published:** 2021-04-28

**Authors:** E Antoun, P Titcombe, K Dalrymple, NT Kitaba, SJ Barton, Ac Flynn, R Murray, ES Garratt, PT Seed, SL White, Cyrus Cooper, H M Inskip, M Hanson, L Poston, KM Godfrey, KA Lillycrop, UPBEAT Consortium/EpiGen Consortium

**Affiliations:** a Human Development and health, Faculty of Medicine, University of Southampton, Southampton, UK; b MRC Lifecourse Epidemiology Unit, University of Southampton, Southampton, UK; c Department of Women and Children’s Health, King’s College London, London, UK; d NIHR, NIHR Southampton BiomedGical Research Centre, Southampton; e Biological Sciences, University of Southampton, Southampton, UK

**Keywords:** Birthweight, dna methylation, pregnancy, early life environment

## Abstract

Many epidemiological studies have linked low birthweight to an increased risk of non-communicable diseases (NCDs) in later life, with epigenetic proceseses suggested as an underlying mechanism. Here, we sought to identify neonatal methylation changes associated with birthweight, at the individual CpG and genomic regional level, and whether the birthweight-associated methylation signatures were associated with specific maternal factors. Using the Illumina Human Methylation EPIC array, we assessed DNA methylation in the cord blood of 557 and 483 infants from the UK Pregnancies Better Eating and Activity Trial and Southampton Women’s Survey, respectively. Adjusting for gestational age and other covariates, an epigenome-wide association study identified 2911 (FDR≤0.05) and 236 (Bonferroni corrected p ≤ 6.45×10−8) differentially methylated CpGs (dmCpGs), and 1230 differentially methylated regions (DMRs) (Stouffer ≤0.05) associated with birthweight. The top birthweight-associated dmCpG was located within the Homeobox Telomere-Binding Protein 1 (HMBOX1) gene with a 195 g (95%CI: −241, −149 g) decrease in birthweight per 10% increase in methylation, while the top DMR was located within the promoter of corticotropin-releasing hormone-binding protein (CRHBP). Furthermore, the birthweight-related dmCpGs were enriched for dmCpGs previously associated with gestational hypertension/pre-eclampsia (14.51%, p = 1.37×10−255), maternal smoking (7.71%, p = 1.50 x 10−57) and maternal plasma folate levels during pregnancy (0.33%, p = 0.029). The identification of birthweight-associated methylation markers, particularly those connected to specific pregnancy complications and exposures, may provide insights into the developmental pathways that affect birthweight and suggest surrogate markers to identify adverse prenatal exposures for stratifying for individuals at risk of later NCDs.

## Introduction

Birthweight can be affected by multiple intrauterine exposures, such as maternal smoking [1,2], maternal body mass index (BMI) [3–5], hypertensive disorders of pregnancy [6,7], gestational diabetes [5,8], and maternal nutrition [9]. All these factors influence foetal growth and, as a consequence, birthweight. Many observational and epidemiological studies have shown a U-shaped relationship between birthweight and an increased risk of later non-communicable diseases (NCDs), including cardio-metabolic disease [10–13], cancer [10,14] and increased mortality [10,15], with babies born at both low and high birthweight being at increased risk. Although a relatively crude marker of foetal growth, birthweight may nonetheless act as a proxy measure for the quality of the intrauterine environment and of detrimental prenatal exposures.

Several mechanisms have been suggested to underlie the associations between birthweight, and by extension the intrauterine environment, and risk of later NCDs, including DNA methylation. A role for DNA methylation in mediating the effects of early life environment on later health is supported by a substantial body of evidence from experimental models [16,17] as well as by large epigenome-wide association studies (EWAS) in humans. The latter have linked adverse early life environments induced by maternal smoking [18,19], dysglycaemia [20,21], hypertension [22] and unbalanced nutrition during pregnancy [23] to altered DNA methylation in neonatal blood and then to increased susceptibility to later NCDs. Consistent with birthweight being a proxy for prenatal exposure to environmental factors, it has also been shown to be associated with neonatal DNA methylation [24]. To date, the majority of the published work on the associations between DNA methylation and birthweight, including the meta-analysis in the PACE consortium by Küpers et al. [24], have been carried out using the Illumina Human Methylation450K array, which measures the methylation at ~450,000 CpGs in the genome [25]. This represents approximately 1.6% of the total CpGs in the genome and was designed to cover regulatory regions involved in transcriptional regulation of gene expression, and as such were located in CpG islands 5ʹUTR, 3ʹUTR and bodies of annotated genes, gene promoters, and a limited number of enhancers. With the updated EPIC array, which measures ~850,000 CpGs, including 90% of the CpGs found on the 450 K array, there is increased coverage across the genome, including a much greater number of probes at regions identified as potential enhancers by both the FANTOM5 [26] and ENCODE [27] projects. Therefore, we sought to investigate the associations between neonatal blood DNA methylation, as measured on the EPIC array, with birthweight. As well as looking at methylation changes at the individual CpG level, we sought to identify differentially methylated regions (DMRs) associated with birthweight. We then investigated the relationships between the birthweight-associated methylation changes and the differentially methylated CpGs (dmCpGs) previously reported to be associated with maternal smoking, obesity, folate levels and pregnancy hypertension/preeclampsia. Finally, we examined whether the methylation at the dmCpGs associated with birthweight may play a functional role by assessing the overlap between these dmCpGs and published cis-expression quantitative trait methylation (cis-eQTMs).

## Materials and Methods

### Study participants

#### UPBEAT – UK Pregnancies Better Eating and Activity Trial

This prospective cohort study was a secondary analysis using data from UPBEAT (isrctn.org registration number 89,971,375). UPBEAT was a multi-centre RCT of a complex dietary and physical activity intervention designed to prevent GDM in obese women and reduce the incidence of large-for-gestational-age (LGA) infants [28]. The cohort comprised 1555 women, older than 16 years of age with a BMI of 30 kg/m^2^ or higher, recruited between 2009 and 2014, who were randomized between 15 weeks 0 days’ and 18 weeks 6 days’ gestation (15^+0^ and 18^+^[6] weeks’ gestation) to either a behavioural intervention superimposed on standard antenatal care or standard antenatal care. As the primary outcomes (GDM and LGA infants) did not differ between intervention and control arms (p = 0.68 and p = 0.40, respectively) [28], all women recruited to the trial were treated as a single cohort for the purposes of this study. Educational attainment was categorized using the highest educational qualification achieved (None, GCE (or equivalent), vocational qualification, A level (or equivalent), first degree, higher degree). Gestational weight gain was characterized as adequate, inadequate or excessive according to the USA National Academy of Medicine 2009 categories (NAM), formerly known as the Institute of Medicine (IOM). [29]. The trial protocol required an Oral Glucose Tolerance Test (OGTT) at 27^+0^ to 28^+^[6] weeks’ but for this study a clinically pragmatic approach was adopted with OGTTs at 23^+^[2] to 30^+0^ weeks’ (mean 27^+^[5]) included. Diagnosis of GDM was according to International Association of Diabetes and Pregnancy Study Groups (IADPSG) criteria (fasting glucose ≥5.1 mmol/l and/or 1 hr ≥10.0 mmol/l and/or 2 hr ≥8.5 mmol/l in response to a 75 g oral glucose load) All aspects of the trial, including the analyses in the present study, were approved by the National Health Service Research Ethics Committee (UK Integrated Research Application System; reference 09/H0802/5) and all participants, including women aged 16 and 17 using Fraser guidelines, provided written informed consent.

#### SWS – Southampton Women’s Survey

The SWS is a prospective mother–offspring cohort study that has assessed the diet, body composition, physical activity, and social circumstances of non-pregnant women aged 20–34 years living in Southampton, UK. Comprehensive details of SWS have been previously published [30]. Educational attainment was categorized using the highest educational qualification achieved (None, CSE, O levels, A levels, Higher national diploma, degree). Gestational weight gain was characterized as adequate, inadequate or excessive according to the USA NAM categories. The SWS cohort did not require an OGTT to be carried out for all participants, therefore, GDM status was obtained from clinical records. Follow-up of the children and sample collection/analysis was carried out under Institutional Review Board approval (Southampton and South West Hampshire Research Ethics Committee, references 276/97, 307/97, 153/99 w, and 10/H0504/30) with written informed consent.

## DNA extraction

Genomic DNA (gDNA) was extracted from the buffy coat of umbilical cord blood samples from UPBEAT using the QIAamp Blood DNA mini kit (Qiagen), and using a standard high salt method for the SWS umbilical cord blood samples. Quality of the genomic DNA was assessed by agarose gel electrophoresis and quantity of gDNA was checked on the NanoDrop ND-1000 (NanoDrop Technologies).

### Infinium Human OmniExpress genotype arrays

SNP genotyping was carried using Human OminiExpress-24v1.2 at the Edinburgh Clinical Research Facility. Genotyping was analysed in Illumina Genome Studio 2.0.2 using genotyping Module 2.0.2 following the manufacturer’s technical note. PLINK version 1.9 beta [31] was used for SNP data management and quality control. Genotyping imputation was carried out using Sanger imputation (https://imputation.sanger.ac.uk). The UK10K [32] and 1000 Genomes Phase 3 [33] reference panels were used with the EAGLE2 imputation pipeline [34,35]. Imputed SNPs were further filtered using imputation info score (INFO >0.8), genotyping call rate 0.05, missingness 0.01, minor allele frequency 0.01, and Hardy-Weinberg equilibrium 1 × 10^−6^. SNPs with a minor allele frequency (MAF) <5% in the whole dataset were excluded from downstream analysis. GEM [36] was used to investigate the genetic influence on methylation.

## Infinium Human MethylationEPIC BeadChip array

DNA methylation using the Infinium Human MethylationEPIC BeadChip array was used to interrogate DNA methylation including 17 technical replicates. 1 µg of the genomic DNA was treated with Sodium Bisulphite using the Zymo EZ DNA Methylation-Gold kit (ZymoResearch, Irvine, California, USA, D5007) and processing of the Human MethylationEPIC (Infinium Methylation 850 K; Illumina, Inc. CA, USA) platform was carried out by the Centre for Molecular Medicine and Therapeutics (CMMT, Canada) (http://www.cmmt.ubc.ca).

## Infinium Human MethylationEPIC BeadChip array data processing

Infinium 850 K data was then processed using the Bioconductor package minfi [37] in R (version 3.4.2). 557 arrays from UPBEAT and 483 arrays from SWS were combined for the purpose of this study with cohort being adjusted for in the analysis (see below). The two cohorts were combined for the initial analysis as power calculations using the pwr package in R calculated that to identify robust associations that pass a Bonferroni adjustment (0.05/775,539) explaining 10% of the variance in birthweight, 758 samples would be required for 90% power. Beta-mixture quantile (BMIQ) normalization was applied to remove array biases and correct for probe design. Probes with a detection p-value >0.01 (n = 18,745) and beadcount <3 (n = 263) were removed from the dataset. CpGs known to cross-hybridize to other locations in the genome [38] (n = 14,759), coinciding with SNPs (n = 77,261), aligning to the sex chromosomes (n = 17,063) and non-CpG probes (n = 2905) were also removed from the dataset. Duplicate samples were included and hierarchical clustering grouped the pairs together. Participant sex was predicted using the getSex() function in minfi. Data was further assessed by visualization of methylation density plots and calculation of median absolute deviation (MAD) scores. Duplicates were removed after normalization but before inference (the duplicate with the lowest MAD score was removed). Eleven samples showed aberrant methylation densities and MAD scores lower than −5 so were removed from the analysis, while nine samples showed aberrant grouping on a multidimensional scaling (MDS) plot, separated by infant sex, and sex discrepencies were removed from subsequent analysis. After excluding preterm births (<37 weeks, n = 35), 985 samples were taken forward for further analysis. ComBat was applied to remove batch (plate) and chip effects [39], and the batch-corrected methylation values used for downstream analysis. The removal of batch effects using ComBat was sufficient to account for the two cohorts used in this study, with PCA analysis showing after BMIQ normalization but before adjustment for chip and cohort of each sample, PC1 separates the two cohorts from each other, while after ComBat the samples are no longer separated by cohort, removing the variation due to cohort from the data (Figure S1). Model assumptions were assessed by visual inspection of QQ plots and p-value histograms and calculation of genomic inflation factor lambda (λ) value, defined as the ratio of the median observed chi squared value distribution to the expected distribution. Genomic inflation (λ) >1.2 was suggestive of test statistic bias and inflation; therefore the bacon package [40] in R was used to reduce this inflation. Raw data used in this analysis has been deposited in the Gene Expression Omnibus (GEO) under accession numbers GSE141065 (UPBEAT) and GSE154915 (SWS).

## Infinium Human MethylationEPIC BeadChip array data analysis

As cord blood comprises a heterogeneous cell population, and no cell composition data was available for these samples, a reference-based prediction of the cell composition was carried out to obtain predicted cellular compositions using the algorithm by Houseman et al. [41], using the FlowSorted.CordBlood.450k [42] package in R, which utilizes the reference for cord blood cell compositions estimated by Andrews and Bakulski [42]. Robust linear regression models using limma [43] were run with methylation as the outcome variable. All models included the following as covariates: Maternal age, maternal smoking during pregnancy, maternal ethnicity, neonate sex, parity, Sentrix position, GDM status (30% of mothers in UPBEAT were diagnosed with GDM by IADSPG criteria [44]), gestational age, and the predicted values for B-cells, CD4 T-cells, CD8 T-cells, granulocytes, monocytes, natural killer cells, and nucleated red blood cell composition. The analysis was controlled for multiple testing with the Benjamini–-Hochberg adjustment for false discovery rate (FDR). Sensitivity analyses were carried out to determine the effect of baseline maternal BMI and educational attainment on any genome-wide methylation changes observed.

## Statistical analysis

All statistical analysis was carried out in R (version 3.4.2). For the purpose of the combined analysis, educational attainment was combined into 5 levels: None, CSE/GCE/O levels, higher national diploma (HND)/vocational qualification, A levels, and Degree. Fisher exact test was used to determine over/under-representation of dmCpGs amongst genomic locations. The hypergeometric distribution probability test was used to determine enrichment of dmCpGs.

## Results

### Participant characteristics

We used data from 995 neonates from the SWS and UPBEAT studies, representing mainly participants of European heritage (SWS = 98.5%, UPBEAT = 73.7%, combined = 85.4%), with a minority of African and Asian ethnicities. There were similar proportions of male and female infants in the two cohorts (52.7% males in the combined dataset). While maternal age and parity were similar in the two cohorts, the average pre-pregnancy BMI in the mothers was higher in UPBEAT (35.2 kg/m^2^ (6.0)) compared to SWS (24.0 kg/m^2^ (5.2)), as was the incidence of GDM (30% of women in the UPBEAT cohort compared to 0.9% in SWS), and maternal smoking (16.6% in UPBEAT, 13.7% in SWS). Birthweight (3522.2 g in SWS infants, 3559 g in UPBEAT infants) was not different between the two cohorts, however, gestational age was slightly less in UPBEAT compared to SWS (39.9 and 40.1 weeks, respectively, p = 0.046).

### Identification of dmCpGs in cord blood associated with birthweight

**Figure 1.**
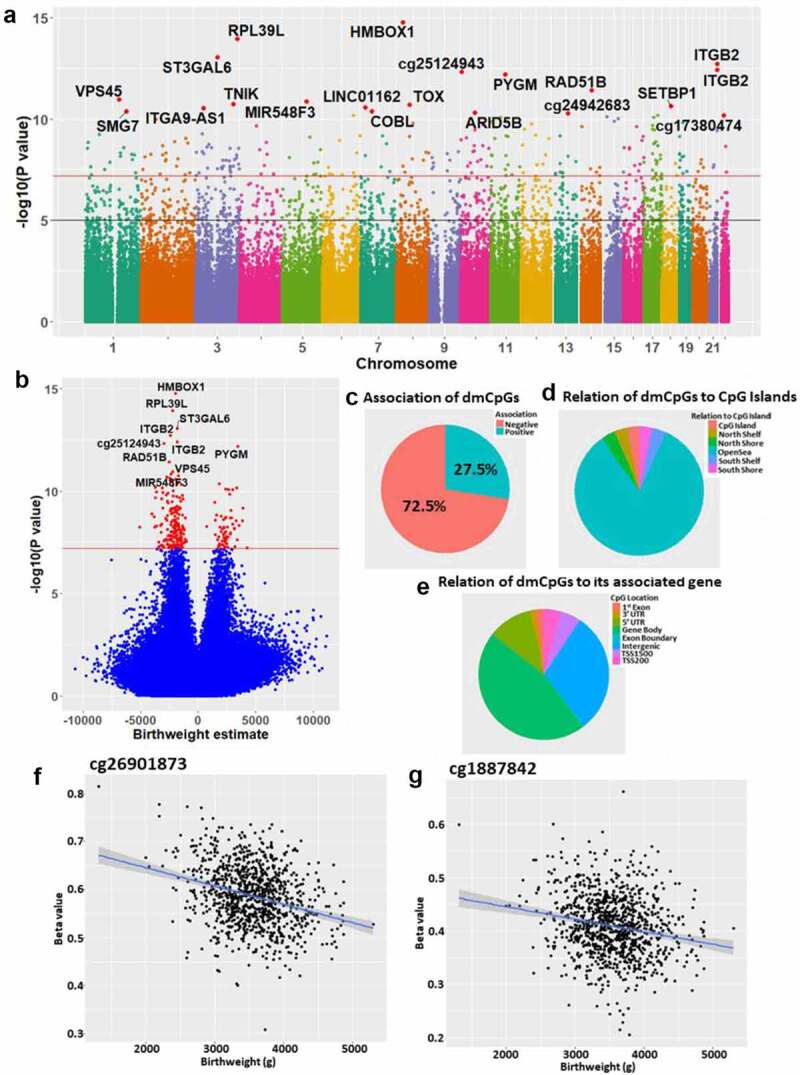
(a) Manhattan plot of the differential methylation results with respect to birthweight, highlighting the genome-wide changes in DNA methylation with respect to birthweight. The black line represents p = 1 x 10^−5^, while the red line represents Bonferroni p = 6.45 x 10^−8^. (b) Volcano plot of the methylation results, with CpGs passing the Bonferroni threshold highlighted in red. The black line represents p = 1 x 10^−5^, while the green line represents Bonferroni p = 6.45 x 10^−8^. (c) Proportion of the Bonferroni dmCpGs showing a negative(red)/positive(blue) association with birthweight. (d) Genomic location of the Bonferroni dmCpGs relative to CpG islands (mainly in open sea – blue). (e) For the dmCpGs associated with a gene, the location of the dmCpG relative to that gene. (F + G) Scatterplots of birthweight (g) and methylation % for the top two birthweight-associated dmCpGs.

DNA from 995 cord blood samples were interrogated for genome-wide DNA methylation levels using the Infinium Human MethylationEPIC BeadChip array. After adjustment for the following covariates; maternal age, GDM status, parity, smoking, ethnicity, neonate sex, gestational age, Sentrix position, and cell-type heterogeneity, methylation levels at 2911 CpGs were found to be associated with birthweight (FDR≤0.05) (Figure 1a+b, Table 2+S1). Of these, 236 CpGs survived the more stringent Bonferroni correction (p < 6.45 x 10^−8^), of which 165 were associated with 152 unique genes. Of the 2911 CpGs with an FDR<0.05, 57.1% showed a negative association with birthweight, while 72.5% of the CpG associations that remained after Bonferroni correction showed a negative association with birthweight (Figure 1c). The top two dmCpGs associated with birthweight following Bonferroni correction were cg26901873 (Bonferroni = 1.30 x 10^−9^, Figure 1f), located in the 5ʹUTR of the Homeobox Telomere-Binding Protein 1 (HMBOX1) gene, which was associated with a 195 g decrease in birthweight for every 10% increase in methylation (95% CI: −241, −149 g, methylation range = 0.308–0.814), and cg18878242 (Bonferroni = 8.93 x 10^−9^, Figure 1g), located in the 5ʹUTR of the Ribosomal Protein L39 like (RPL39L) gene, associated with a 222 g decrease in birthweight for every 10% increase in methylation (95% CI: −274, −170 g, methylation range = 0.206–0.661). cg08801887, located in the body of the T Cell Immune Regulator 1, ATPase H+ Transporting V0 Subunit A3 (TCIRG1) gene, had the largest positive association, with a 10% increase in methylation associated with a 422 g increase in birthweight (95% CI: 275, 569 g, methylation range = 0.626–0.911). cg07749613, located in the intergenic region on chromosome 2 had the largest negative association, with a 10% increase in methylation associated with a 507 g decrease in birthweight (95% CI: −685, −330 g, methylation range = 0.021–0.164).Table 1

**Table 1.** Participant characteristics

		**SWS** **(n = 460)**	**UPBEAT** **(n = 525)**	**Combined** **(n = 985)**
**Neonate Sex**	**Male (%)**	52.0	53.2	52.7
**Maternal ethnicity**	**White (%)**	98.5	73.7	85.4
	**Asian (%)**	0.0	5.3	2.8
	**Black (%)**	1.1	16.4	9.2
	**Other (%)**	0.4	4.6	2.6
**Educational attainment** *(4 missing)*	**None (%)**	1.3	1.9	1.6
	**CSE/O levels/GCE (%)**	34.1	13.9	23.4
	**HND/Vocational (%)**	8.1	24.8	17.0
	**A levels (%)**	31.3	15.5	22.9
	**Degree (%)**	25.3	43.9	35.1
**IOM weight gain category** *(49 missing)*	**Adequate (%)**	28.4	37.1	33.0
	**Inadequate (%)**	22.0	29.3	25.9
	**Excessive (%)**	49.7	33.7	41.1
**GDM** *(8 missing)*	**Yes (%)**	0.9	30.0	16.1
**Parity**	**Primiparous (%)**	50.96	52.29	51.7
**Smoking**	**Smoker (%)**	13.7	16.6	14.97
**Birthweight (g)**† *(2 missing)*		3523.2 ± 490.3	3559.1 ± 469.0	3542.1 ± 479.3
Maternal BMI (kg/m^2^)*		24.0 (5.2)	35.2 (6.0)	32.0 (11.7)
**Maternal Age (years)**†		31.2 ± 3.6	30.9 ± 5.4	31.1 ± 4.7
**Gestational age (weeks)**†		40.1 ± 1.2	39.9 ± 1.3	40.0 ± 1.3
**B cell**†		0.08 ± 0.02	0.11 ± 0.05	0.10 ± 0.04
**CD4 T cells**†		0.12 ± 0.06	0.13 ± 0.08	0.13 ± 0.07
**CD8 T cells**†		0.14 ± 0.04	0.12 ± 0.04	0.13 ± 0.04
**Granulocytes**†		0.50 ± 0.09	0.45 ± 0.13	0.48 ± 0.11
**Monocytes**†		0.09 ± 0.02	0.10 ± 0.04	0.10 ± 0.04
**Natural Killer cells**†		0.01 ± 0.01	0.02 ± 0.03	0.01 ± 0.02
**Nucleated Red Blood Cells**†		0.10 ± 0.06	0.10 ± 0.07	0.10 ± 0.06

*median (IQR), †mean±sd, CSE = Certificate of Secondary Education, GCE = General Certificate of Education, O level = Ordinary level, HND = Higher National Diploma, A level = Advanced level, IOM = Institute of Medicine, GDM = Gestational Diabetes Mellitus, BMI = Body Mass Index

**Table 2.** Top 20 dmCpGs associated with birthweight

**CpG**	**Estimate**	**FDR**	**Bonferroni**	**hg19 coordinates**	**Gene**	**Gene location**	**Relation to CpG Island**
cg26901873	−1949.77	1.30E-09	1.30E-09	chr8:28,811,004-	HMBOX1	5ʹUTR	OpenSea
cg18878242	−2220.21	4.47E-09	8.93E-09	chr3:186,848,672+	RPL39L	5ʹUTR	OpenSea
cg19274030	−1809.59	2.28E-08	6.84E-08	chr3:98,489,745+	ST3GAL6	5ʹUTR	OpenSea
cg24797865	−2444.55	3.64E-08	1.45E-07	chr21:46,331,470+	ITGB2	5ʹUTR	OpenSea
cg16015397	−1806.24	6.02E-08	3.03E-07	chr21:46,331,472+	ITGB2	5ʹUTR	OpenSea
cg25124943	−2974.16	6.02E-08	3.61E-07	chr10:4,117,248+			OpenSea
cg16058496	3435.52	7.34E-08	5.14E-07	chr11:64,527,499+	PYGM	1stExon	OpenSea
cg00464852	−2500.34	3.73E-07	2.98E-06	chr14:68,610,155+	RAD51B	Body	OpenSea
cg18001737	−2230.54	9.65E-07	8.68E-06	chr1:150,081,706+	VPS45	Body	OpenSea
cg19152802	−2355.45	1.10E-06	1.10E-05	chr5:109,849,887-	MIR548F3/TMEM232	TSS1500/Body	OpenSea
cg09782624	−1721.53	1.26E-06	1.39E-05	chr3:170,908,539-	TNIK	Body	OpenSea
cg20407747	−2708.45	1.29E-06	1.55E-05	chr8:59,941,881-	TOX	Body	OpenSea
cg21180953	−2464.28	1.36E-06	1.77E-05	chr18:42,489,607-	SETBP1	Body	OpenSea
cg10777338	−1995.49	1.45E-06	2.03E-05	chr7:20,936,700+	LINC01162	Body	OpenSea
cg12732215	−2381.16	1.52E-06	2.28E-05	chr3:37,795,217-	ITGA9-AS1/ITGA9	Body	OpenSea
cg00506306	−1809.93	1.99E-06	3.32E-05	chr7:51,207,974-	COBL	Body	OpenSea
cg13492133	1841.23	1.99E-06	3.37E-05	chr1:183,516,266+	SMG7	Body	OpenSea
cg25953130	−1468.10	2.08E-06	3.75E-05	chr10:63,753,550+	ARID5B	Body	OpenSea
cg24942683	−2207.16	2.12E-06	4.02E-05	chr13:77,518,644-			OpenSea
cg17380474	−3704.65	2.39E-06	5.04E-05	chr22:30,609,292-			OpenSea

Of the dmCpGs remaining after Bonferroni correction, there was an over-representation of CpGs in the OpenSea regions (odds ratio = 3.95, p = 4.99 x 10^−19^) compared to a significant under-representation of CpGs in islands (odds ratio = 0.13, p = 1.83 x 10^−13^) and shores (odds ratio = 0.35, p = 252 x 10^−6^)(Figure 1d). Of the 165 dmCpGs that were associated with a gene, there was a significant underrepresentation of CpGs at the transcription start site of genes (odds ratio = 0.45, p = 1.42 x 10^−4^)(Figure 1e).

### Sensitivity analysis

As a number of maternal factors such as maternal BMI [45,46], gestational weight gain [47], and educational attainment [48,49] have previously been shown to be associated with birthweight, we carried out a series of sensitivity analyses, analysing DNA methylation with respect to birthweight with adjustment for each of these factors in the original model as additional covariates. After the addition of maternal BMI into the regression model, 2911 CpGs showed a significant association with child’s birthweight (FDR<0.05, table S2)), of which 243 survived the more stringent Bonferroni correction for multiple comparisons; there was a 91.6% overlap amongst the dmCpGs with an FDR <0.05 from the original analysis, and a 53.91% overlap amongst the Bonferroni adjusted birthweight dmCpGs. Of the dmCpGs with an FDR<0.05, there was also a strong correlation between the effect estimates of the birthweight associated CpGs (Pearson correlation coefficient = 0.999, ICC = 0.999, p < 2.2 x 10^−16^, figure S2a). After the addition of gestational weight gain (GWG) into the original regression model, 1195 dmCpGs were associated with birthweight (FDR<0.05, Table S3), of which 87 remained after the more stringent Bonferroni adjustment. All 87 of the Bonferroni adjusted and 98.3% of the FDR<0.05 dmCpGs in GWG adjusted analysis were also significantly associated with birthweight in the main analysis. Of the dmCpGs with an FDR<0.05, there was also a strong correlation between the effect estimates of the birthweight associated CpGs (Pearson correlation coefficient = 0.997, ICC = 0.996, p < 2.2 x 10^−16^, figure S2b). Furthermore, when the model was additionally adjusted for maternal educational attainment, 2922 CpGs showed a significant association with child’s birthweight (FDR<0.05, Table S4), of which 247 remained after the more stringent Bonferroni correction for multiple comparisons, with a 51.42% overlap between the Bonferroni adjusted dmCpGs and a 92.20% overlap amongst the FDR<0.05 dmCpGs; there was also a strong correlation between the effect estimates of the birthweight associated CpGs with a FDR<0.05 (Pearson correlation coefficient = 0.999, ICC = 0.999, p < 2.2 x 10^−16^, figure S2c).

When restricting the analysis to participants of European descent, the findings were consistent with the main analysis; 73.6% of the CpGs with an FDR<0.05 in the restricted analysis on participants of European descent were found to be associated with the birthweight dmCpGs in the main analysis on all participants, with a strong correlation between the effect estimates of the birthweight-associated CpGs of the two analyses (Pearson correlation coefficient = 0.996, ICC = 0.991, p < 2.2 x 10^−16^, figure S2d, Table S5). Similarly, when restricting the correlation to the 236 CpGs from the main analysis that survive Bonferroni correction, there was a strong correlation between the effect estimates between the main analysis and the restricted analysis on participants of European descent (Pearson correlation coefficient = 0.998). Due to the low number of African and Asian participants in this data set (UPBEAT n = 90 and SWS n = 25), it was not possible to repeat the analysis restricted to these populations. The findings were also consistent when neonates who were born preterm were included, with 99% of the original birthweight associated dmCpGs (at both FDR and Bonferroni <0.05, Figure S2b+d, Table S6) also being associated with birthweight when preterm infants were included. There was also a strong correlation between the effect estimates of the birthweight associated CpGs (Pearson correlation coefficient = 0.992, ICC = 0.98, p = p < 2.2 x 10^−16^, figure S2e).

### Overlap between the birthweight-associated dmCpGs in the UPBEAT and SWS cohorts

**Figure 2.**
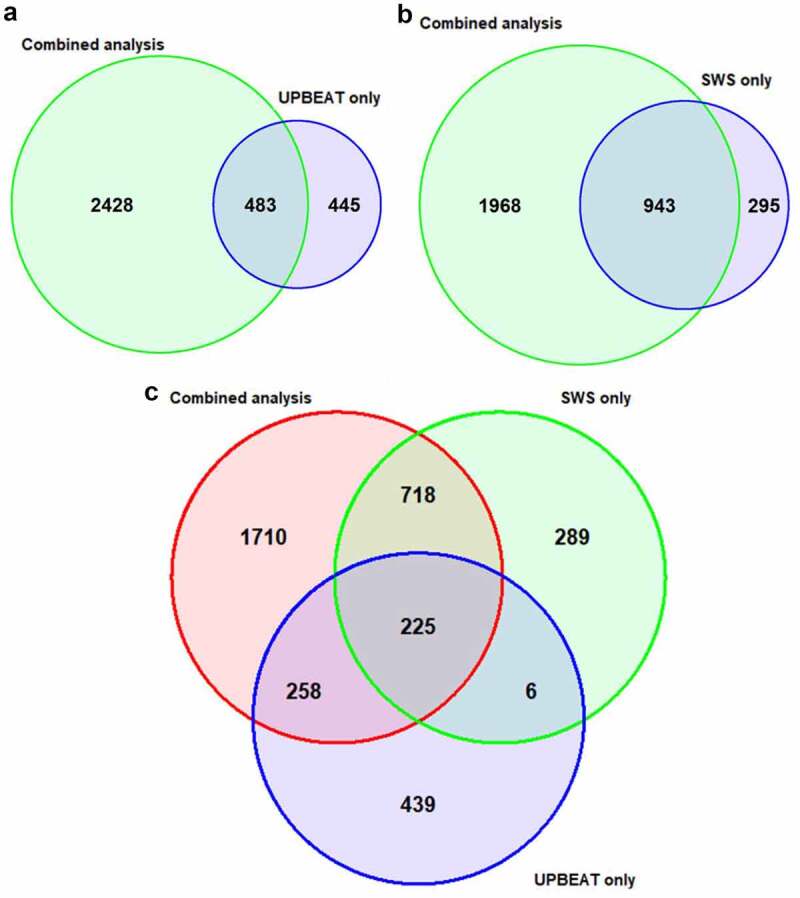
Overlap of the birthweight-associated dmCpGs (FDR<0.05) in the main combined analysis and in the (a) UPBEAT only analysis and (b) the SWS only analysis. (c) Three-way overlap between the dmCpGs in the combined analysis, UPBEAT only analysis and the SWS analysis. All show high overlap between the dmCpGs in the different analysis, with 255 dmCpGs (FDR<0.05) identified in the combined, UPBEAT only and SWS only analysis.

As the main analysis was based on combining data from the SWS and UPBEAT cohorts, we also analysed the two cohorts separately. In the SWS only analysis, there were 1238 birthweight-associated dmCpGs (FDR<0.05), of which 76.2% overlapped with the main analysis, while in the UPBEAT only analysis, of the 928 birthweight-associated dmCpGs (FDR<0.05), 52.0% overlapped with the main analysis (Figure 2, Table 3). Comparison of the overlap between the single cohort analyses revealed 33.1% of the dmCpGs in the UPBEAT single cohort analysis were also differentially methylated in the SWS single cohort analysis, of which 97.4% were significantly associated with birthweight in the main analysis (Figure 2c).

**Table 3.** Top 10 birthweight-associated dmCpGs in the separate SWS and UPBEAT analyses

**CpG**	**Estimate**	**FDR**	**hg19 coordinates**	**Gene**
**SWS only**				
cg15908709	5209.34	1.37E-06	chr17:46,676,215-	LOC404266/HOXB6
cg16964184	−2884.79	2.84E-06	chr10:91,411,848-	
cg13245626	−2259.42	3.22E-06	chr4:75,558,301+	
cg16058496	5535.96	3.22E-06	chr11:64,527,499+	PYGM
cg25017876	−2726.07	2.11E-05	chr10:98,066,585-	DNTT
cg07624582	−2946.44	2.11E-05	chr9:14,271,314+	NFIB
cg17254383	−5326.22	2.11E-05	chr17:53,363,128-	HLF
cg26240885	−2500.99	2.11E-05	chr12:15,055,618+	
cg24020157	−3782.24	2.11E-05	chr10:43,697,521+	RASGEF1A
**UPBEAT only**				
cg23638139	2843.94	4.73E-08	chr11:73,104,087+	RELT
cg17264028	2554.18	2.34E-04	chr2:73,487,972-	FBXO41
cg18402166	830.03	2.34E-04	chr17:62,778,279-	LOC146880
cg07703979	2017.70	3.74E-04	chr1:53,579,547+	SLC1A7
cg07977153	3049.19	3.74E-04	chr11:1,967,958+	MRPL23
cg25124943	−1585.49	3.74E-04	chr10:4,117,248+	
cg16708012	1696.31	3.82E-04	chr12:108,992,114+	TMEM119
cg24276069	2278.11	5.10E-04	chr1:45,243,927+	RPS8/SNORD38B
cg10163377	1645.26	5.18E-04	chr10:99,478,368-	LOC100270710
cg13638867	1071.26	5.69E-04	chr11:2,241,568-

### Differentially methylated regions in the infants’ methylome are associated with birthweight

Regional analysis of differential methylation associated with birthweight identified 1250 DMRs with a Stouffer<0.05 (Table 4 and S7). The DMRs were spread throughout the genome, and not localized to a particular region, with 41.8% of the significant DMRs showing greater methylation with increased birthweight. The top DMR was located in the promoter region of the corticotropin-releasing hormone-binding protein (CRHBP), consisting of 11 CpGs spanning a region of 1448bp (Stouffer = 3.05 x 10^−24^). Of the 1250 birthweight-associated DMRs, 40 contained a dmCpG (Bonferroni<0.05) previously reported by Küpers et al. [24] to be associated with birthweight, with a further 28 DMRs associated with the same gene for which a dmCpG had previously been reported [24].

**Table 4.** Top 20 birthweight-associated DMRs identified by DMRcate

**Chr**	**Start**	**End**	**Width**	**No. CpGs**	**Stouffer**	**Overlapping promoters**
chr5	76,247,647	76,249,094	1448	11	3.05E-24	CRHBP
chr11	2,011,216	2,011,346	131	6	1.62E-21	AC051649.6/MRPL23-AS1
chr19	940,724	941,571	848	5	4.90E-21	
chr13	51,417,469	51,418,614	1146	12	4.12E-18	DLEU7
chr3	155,421,735	155,422,457	723	8	1.57E-16	PLCH1
chr17	75,537,017	75,537,444	428	3	4.86E-15	
chr15	74,494,515	74,496,040	1526	16	2.37E-14	STRA6/RP11-60L3.1
chr16	68,321,421	68,321,730	310	6	4.27E-14	SLC7A6
chr19	1,074,425	1,075,104	680	4	6.38E-14	HMHA1
chr17	907,643	907,886	244	4	7.83E-14	
chr17	47,076,904	47,077,165	262	2	2.23E-13	IGF2BP1
chr14	22,902,226	22,902,405	180	3	4.25E-13	AE000661.37
chr20	36,024,542	36,024,669	128	5	4.45E-13	SRC
chr6	139,454,751	139,454,928	178	3	1.21E-12	HECA
chr16	89,734,986	89,735,184	199	2	3.16E-12	
chr11	64,527,240	64,527,846	607	8	4.11E-12	PYGM
chr11	44,642,868	44,642,932	65	3	5.61E-12	
chr20	62,687,969	62,688,896	928	12	6.42E-12	TCEA2/RP13-152O15.5
chr21	46,330,726	46,331,472	747	6	9.82E-12	ITGB2
chr9	18,260,702	18,260,848	147	2	1.93E-11	

### Functional analysis

To determine whether there were any gene ontology (GO) terms enriched amongst the birthweight-associated 2911 dmCpGs (FDR<0.05), gene ontology enrichment analysis was carried out using the missMethyl package [50] in R. We found enrichment of genes associated with 3 GO terms: GO:0032501: multicellular organismal process (FDR = 0.032); GO:0051239, regulation of multicellular organismal process (FDR = 0.041); and GO:0048513, animal organ development (FDR = 0.049). There were no KEGG terms enriched amongst the birthweight-associated dmCpGs.

To determine whether the birthweight-associated dmCpGs showed a functional correlation with gene expression, we compared our list of 1206 dmCpGs, the sites found on both the 450 K and 850 K arrays (FDR<0.05), with a published list of 18,881 cis-expression quantitative trait methylation (cis-eQTM) [51], CpG sites known to correlate with gene expression from adult whole blood samples. Of the 1206 dmCpGs, 93 were reported to be a cis-eQTM, associated with 123 genes (enrichment p-value = 4.439 x 10^−6^).

### Maternal factors

A meta-analysis has been previously carried out by the PACE consortium to determine the effect of maternal factors, including smoking during pregnancy [18], BMI [22], obesity [22], folate levels [52], and gestational hypertension/pre-eclampsia [53], on the DNA methylation of the offspring at birth. These analyses were carried out using the 450 K array. Restricting the birthweight-associated dmCpGs (FDR<0.05) from our study to those CpGs present on the 450 K array (1206/2911), we investigated whether the birthweight-associated dmCpGs in this study were enriched amongst the dmCpGs found to be associated with a range of early life environmental factors by the PACE consortium study in their meta analysis.

There was a significant enrichment of the birthweight-associated dmCpGs in our study with the PACE study dmCpGs associated with gestational hypertension/pre-eclampsia (14.51%, p = 1.37 x 10^−255^), maternal smoking (7.71%, p = 1.50 x 10^−54^) and maternal plasma folate levels during pregnancy (0.33%, p = 0.029). Meta-analyses with respect to maternal BMI and obesity have only to date shown CpGs that pass Bonferroni correction; comparing these dmCpGs to those identified in our study we found no evidence of enrichment of maternal BMI-associated CpGs amongst the birthweight-associated dmCpGs. Similarly, restricting the analysis to only the birthweight-associated dmCpGs that remain after Bonferroni correction, we found a significant enrichment of CpGs associated with gestational hypertension/pre-eclampsia (13.33%, p = 1.25 x 10^−34^) and maternal smoking (7.78%, p = 1.44 x 10^−10^), but not with maternal BMI or plasma folate levels during pregnancy, BMI, or obesity.

### Metastable epialleles

We next tested the birthweight-associated dmCpGs for enrichment of metastable epialleles [54,55]. We found no evidence of enrichment of the birthweight-associated dmCpGs for metastable epialleles, with only 10/3840 reported metastable epialleles overlapping one of the 2911 birthweight-associated dmCpGs (p = 0.91).

### Influence of genetic variation on the birthweight-associated dmCpGs

As DNA methylation can be driven by genotype and act as an integrator of the individual’s genotype and environmental exposure, we investigated the potential effect of genotype on the birthweight-associated dmCpGs (Bonferroni<0.05) and any interaction between genetic variation and birthweight, by carrying out a genome-wide mQTL screen using the GEM package. 9109 significant mQTLs (FDR<0.05, table S8) were identified, with 224 of the birthweight-associated dmCpGs significantly associated with the genotype at one of 8506 unique SNPs. 1153 of the mQTLs identified were cis-mQTLs, while 7956 were trans-mQTLs. Of these mQTLs, 95 have been previously reported in the ARIES mQTL dataset [56] in cord blood at birth.

**Figure 3.**
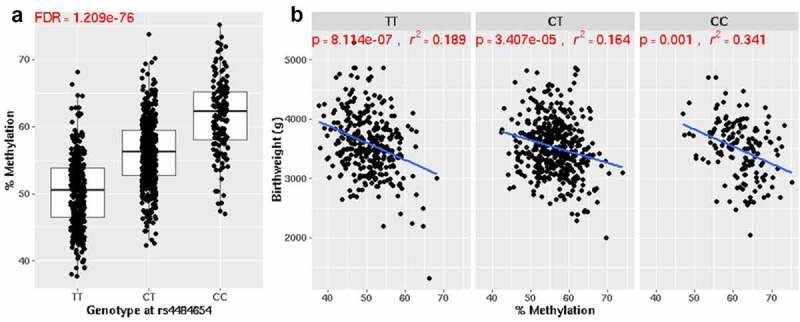
(a) The top mQTL identified was with cg14908202, located in body of the ADAM5 gene and rs4484654. There was an increase in methylation level at the CpG with each extra copy of the alternative (‘C’) allele at this position. (b) Despite rs4484654 affecting methylation levels at cg14908202, the association between this CpG and birthweight remain significant regardless of genotype at this SNP.

The top mQTL identified was rs4484654, with each copy of the alternative C allele at this position being associated with an increase in methylation level at cg14908202, located in the body of the ADAM5 gene (Figure 3a). Despite rs4484654 affecting DNA methylation levels at cg14908202, the association between DNA methylation at cg14908202 and birthweight remained significant after stratification by genotype at rs4484654 (Figure 3b), or when genotype was included as a covariate in the regression model (p = 8.09 x 10^−11^). Similarly, the association between the remaining 221 dmCpGs, for which genotype was shown to influence DNA methylation, and birthweight remained after the inclusion of the relevant mQTLs as a covariate in the regression models.

## Discussion

In this epigenome-wide study, using the Human MethylationEPIC array, we report novel neonatal DNA methylation signatures associated with birthweight. Moreover, the birthweight-associated dmCpGs were enriched amongst previously published cis-eQTMs, CpG loci known to correlate with gene expression, as well as amongst genes associated with developmental pathways, potentially suggesting a functional role for these dmCpGs in the offspring. The birthweight-related dmCpGs were also enriched for CpGs sites previously linked to maternal smoking during pregnancy, hypertension/pre-eclampsia, and maternal folate levels, suggesting that early life environmental factors and stressors during pregnancy may be important determinants of neonatal DNA methylation and potentially contribute to variations in birthweight. The identification of birthweight-associated methylation markers, particularly those connected to specific pregnancy complications and exposures, may provide valuable insights into the developmental pathways that affect birthweight and allow the identification of methylation signatures associated with adverse prenatal exposures to stratify individuals at risk of later NCDs.

Birthweight was associated with changes in neonatal DNA methylation at both the CpG and regional level, with dmCpGs being enriched amongst open seas and, when associated with a gene, under-represented within the transcriptional start site (TSS), suggesting enrichment of dmCpGs within enhancer or long range regulatory regions. Moreover, 93 of the 1206 dmCpGs (these were the birthweight-associated dmCpGs covered on both the 450 K and 850 K arrays) were reported to be a cis-eQTM, and associated with 123 genes, suggesting that differential methylation at these sites may be associated with functional changes in gene expression. In this study, we only investigated the overlap between the birthweight-associated dmCpGs and reported cis-eQTMs. Although the majority of our dmCpGs were not reported to be cis-eQTMs, a large proportion of the dmCpGs were found to be associated with trans-mQTLs. Bonder at al. [51] reported that trans-mQTL CpGs are enriched around TSSs and within Hi-C interchromosomal contacts, overlapping the binding sites of transcription factors that regulate chromatin architecture (e.g., CTCF, RAD21). This suggests that although the majority of the dmCpGs are not direct eQTMs, they may play a role in regulating chromatin architecture, which in turn regulates transcription and gene expression.

A number of previous studies, including the meta-analysis by the PACE consortium [24], have reported changes in neonatal DNA methylation signatures with respect to birthweight using the 450 K array. Of the 1206 birthweight associated dmCpGs identified in this study that were also present on the 450 K array, 415 (34.41%) were sites identified previously by Küpers et al. to be associated with birthweight, suggesting that there are methylation signatures associated with birthweight that are robustly replicated across cohorts. However, 58% of the 2911 birthweight-associated dmCpGs (FDR<0.05) identified in this present study were unique to the 850 K array, consistent with the enrichment of the birthweight associated dmCpGs to regulatory or enhancer regions, regions with enhanced coverage on the 850 K array. The top dmCpG associated with birthweight in this study was HMBOX1. This CpG is not present on the HumanMethylation 450 K array. HMBOX1 encodes a transcriptional repressor [57], which has been reported to play roles in the differentiation of bone marrow stromal cells (BMSCs), and embryonic stem cells to endothelial cells [58,59], inhibition of apoptosis [60] and telomere maintenance [61]. A dmCpG within the body of the T Cell Immune Regulator 1, ATPase H+ Transporting V0 Subunit A3 (TCIRG1) gene was associated with the largest change in birthweight. This gene is a member of a family of ATP-dependent proton pumps that are responsible for the acidification of intracellular compartments involved in processes, such as receptor-mediated endocytosis [62]. TCIRG1 and HMBOX1 have not previously been linked to birthweight so whether they are functionally involved in foetal growth, or simply markers of altered birthweight is unknown. The top DMR associated with birthweight was located within CRHBP. Eight out of the eleven CpGs in the CRHBP DMR are not present on the 450 K array. Of the three that are on the 450 K array, one was identified by Kupers et al [24]. CRHBP encodes for the corticotropin-releasing hormone-binding protein, which inactivates corticotropin-releasing hormone (CRH) and has been suggested to be involved in regulating foetal pituitary-adrenal function; in humans, placental CRH is known to play an important role in determining the length of gestation and the timing of parturition [63] and has been implicated in low birthweight, with higher placental CRH production in mid-gestation shown to predict lower birthweight [63]. More recently, SNPs within CRHBP have been shown to be associated with variations in birthweight in three ethnically diverse populations [64]. The differential methylation of this gene suggests that not only genetic but also epigenetic regulation of this gene and hence the CRH pathway may be an important determinant of foetal growth and birthweight, via the foetal pituitary ACTH and adrenal cortisol secretion.

Maternal factors have been strongly linked to DNA methylation changes in the infant. Interestingly, there was no overlap amongst the birthweight-associated dmCpGs with metastable epialleles suggesting that the factors that influence birthweight and associated methylation patterns operate throughout pregnancy, rather than in the very earliest stages of pregnancy. Indeed we found that the birthweight-associated dmCpGs were enriched for CpGs previously identified by the PACE consortium to be associated with maternal hypertension [53], smoking during pregnancy [18] and folate status [52] suggesting that a proportion of the dmCpGs, and birthweight itself, may be driven by these maternal factors. Of the dmCpGs enriched amongst the smoking, hypertension, or folate associated CpGs, the majority were only associated with one of these maternal factors, suggesting specificity of effect. There was, however, no enrichment amongst the birthweight-associated dmCpGs with CpGs associated with maternal BMI [22]. This may be because the analysis was restricted to the 450k birthweight-associated CpGs identified by the PACE consortium analysis, however, adjustment for maternal BMI in our analysis had minimal effect on the number or location of the significant dmCpGs, with over 90% of dmCpGs in the adjusted analyses being present in the unadjusted analyses. Furthermore, comparison of the birthweight-associated dmCpGs in the combined cohort analysis with those associated with birthweight in the UPBEAT cohort alone showed a considerable overlap between the two analyses, even though all women in the UPBEAT cohort had a BMI of over 30, consistent with the majority of identified dmCpGs being largely independent of maternal BMI. It has been suggested that maternal BMI may programme offspring adiposity through birthweight and changes in the foetal epigenome [65–67]. However, the finding that the birthweight associated methylation changes are not related to previously reported methylation changes associated with maternal BMI suggests either the maternal BMI/birthweight/offspring adiposity axis is unrelated to methylation, or birth weight per se is not the mediator. Consistent with this, recent mediation analyses have shown that most of the effect of pre-pregnancy obesity on childhood weight-related anthropometric outcomes is not mediated through offspring’s birthweight [68,69] .

DNA methylation can be driven by both the environment and genotype and, while we found that the methylation status of the majority of the birthweight associated dmCpGs were associated with genotype, only a minority of these showed a significant interaction with birthweight and genotype. Further large studies will be required to determine the precise contribution that both genotype and early life environmental factors make to these methylation changes and their impact on birthweight.

A strength of this study is that we analysed DNA methylation on the 850 K platform rather than the 450 K platform, giving a far greater coverage of gene regulatory regions which may have functional significance. Moreover, by combining the analysis for birthweight across the SWS and UPBEAT cohorts, the analysis included infants born to women across a much wider range of body mass indices, from 17 to 59 kg/m^2^. The majority of studies to date have used cohorts consisting solely of normal weight individuals, excluding individuals with a BMI>30 kg/m^2^. Importantly, we show that although the birthweight-related methylation signatures were associated with a number of maternal environmental factors, they were largely independent of maternal BMI.

There are some limitations to this study. Firstly we analysed DNA methylation in cord blood and the functional consequences of altered methylation at such marks are unknown. However, a number of the dmCpGs were eQTMs, suggesting that at least some of the changes in DNA methylation are associated with a change in gene expression. Secondly, although we found that many of the birthweight-associated dmCpGs were also associated with CpGs previously shown to be associated with specific maternal factors, whether these dmCpGs play a causal role in mediating the effect on birthweight or are simply markers of the change in birthweight is unknown. Even if they are markers of birthweight rather than mechanistically involved in affecting foetal growth, they may still be useful in identification of risk or exposures. The third limitation is the relatively modest sample size, although we did find robust associations with birthweight which passed a stringent Bonferroni correction, and there was considerable overlap with the meta-analysis of birthweight-associated dmCpGs previously found using the 450 K array [24].

## Conclusion

Here we show that birthweight is associated with widespread changes in DNA methylation. These were for the most part independent of genotype and enriched for CpGs associated with a range of environmental exposures including maternal smoking during pregnancy, hypertension/preeclampsia and folate status, supporting the concept that early life environment is an important determinant of variations in birthweight. The identification of birthweight-associated methylation markers, particularly those associated with specific maternal factors, may provide valuable insights into the developmental pathways involved [70] and/or act as markers to identify adverse exposures and which could be used to stratify individuals at risk.

## Supplementary Material

Supplemental Material
