# Supplementary material for: DNA methylation signatures in cord blood associated with birthweight are enriched for dmCpGs previously associated with maternal hypertension or pre-eclampsia, smoking and folic acid intake
Source: Epigenetics. 2021 Apr 28;17(4):405–21. doi: 10.1080/15592294.2021.1908706 (PMC8993070; doi:10.1080/15592294.2021.1908706)
Supplement: Supplemental Material [file KEPI_A_1908706_SM2137.rtf]

Figure S1: (A) PCA plots of the data after BMIQ normalization but before adjustment for chip and cohort of each sample. As expected PC1 separates the two cohorts from each other, suggesting the majority of the variation in the beta values are due to the cohort each sample has come from. (B)  PCA plot of the data after adjustment for chip and cohort of each sample using ComBat from the 'sva' package in R. The samples are no longer separated by cohort, removing the variation due to chip and cohort from the data. 
Figure S1:


Figure S2: Bland-Altman plots for the difference in the estimates of the dmCpGs (FDR<0.05) (A) in the main analysis and analysis with additional adjustment for maternal BMI, (B) in the main analysis and analysis with additional adjustment for maternal gestational weight gain, (C) in the main analysis and analysis with additional adjustment for maternal educational attainment, (D) in the main analysis and analysis with participants from European descent only and (E) in the main analysis and analysis with preterm births included (gestational age < 37 weeks). ICC=intraclass correlation coefficient
Figure S2:
